# Gut–Liver Axis Failure in Critical Alcohol‐Associated Liver Disease: From ICU Secondary Hits to Microbiome‐Targeted Therapy

**DOI:** 10.1155/mi/3968719

**Published:** 2026-05-31

**Authors:** Yuting Zhang, Yiyu Wang, Yong Yang, Hong Mei, Xinxin Liu, Yuanxiu He, Song Qin, Banghai Feng

**Affiliations:** ^1^ Department of Critical Care Medicine, Affiliated Hospital of Zunyi Medical University, Zunyi, 563000, Guizhou, China, zmchospital.com.cn; ^2^ Department of Critical Care Medicine, Kweichow Moutai Hospital, Renhuai, 564500, Guizhou, China; ^3^ Department of Anesthesia and Perioperative Care, UCSF School of Medicine, San Francisco, 94158, California, USA, ucsf.edu; ^4^ Department of Critical Care Medicine, Zunyi Hospital of Traditional Chinese Medicine, Zunyi, 563000, Guizhou, China

**Keywords:** alcohol-associated liver disease, gut–liver axis, intensive care, intestinal barrier dysfunction, microbiome-targeted therapy, multiple organ dysfunction

## Abstract

Alcohol‐associated liver disease (ALD) can progress to critical illness phenotypes requiring intensive care, including severe alcohol‐associated hepatitis, acute decompensation, and alcohol‐associated acute‐on‐chronic liver failure (ACLF). In these patients, short‐term outcomes are driven less by the burden of fibrosis alone than by systemic inflammation, immune dysfunction, infection, and multiorgan failure. At the core of this process is gut–liver axis failure, which links alcohol‐induced dysbiosis and intestinal barrier disruption to microbial translocation, hepatic innate immune activation, and systemic inflammatory amplification. In the intensive care unit (ICU), secondary hits such as broad‐spectrum antibiotics, acid suppression, parenteral nutrition, shock, sedatives or opioids, and mechanical ventilation may further exacerbate these mechanisms and disturb microbial ecology and barrier integrity. Microbiome‐targeted therapies (probiotics, postbiotics, and fecal microbiota transplantation) are biologically plausible. However, current evidence is mainly derived from non‐ICU or relatively stable ALD populations. Therefore, their use in critically ill patients requires strict safety boundaries, including severe barrier disruption, invasive devices, uncontrolled infections, and profound immune dysfunction. This narrative review synthesizes the pathophysiological continuum from gut barrier failure to systemic inflammation and multiorgan dysfunction in critical ALD, with particular emphasis on ICU‐specific secondary hits, safety‐aware microbiome modulation, and future phenotype‐informed precision strategies.

## 1. Introduction

Alcohol‐associated liver disease (ALD) is a leading cause of chronic liver injury worldwide, and its severe phenotypes, including severe alcoholic hepatitis (AH), acute decompensation, and acute‐on‐chronic liver failure (ACLF), often require admission to the intensive care unit (ICU). In these critically ill patients, short‐term outcomes are determined less by the rate of fibrosis progression than by acute systemic inflammation, immune dysfunction, infection, and multiorgan failure [1, 2]. Gut–liver axis dysfunction has been established as a central amplifier of this process. Alcohol‐induced dysbiosis and disruption of intestinal barrier integrity promote microbial translocation to the liver, triggering hepatic innate immune activation and the release of potent inflammatory mediators, which together drive systemic inflammation and multiorgan dysfunction [3, 4].

**Figure 1:**
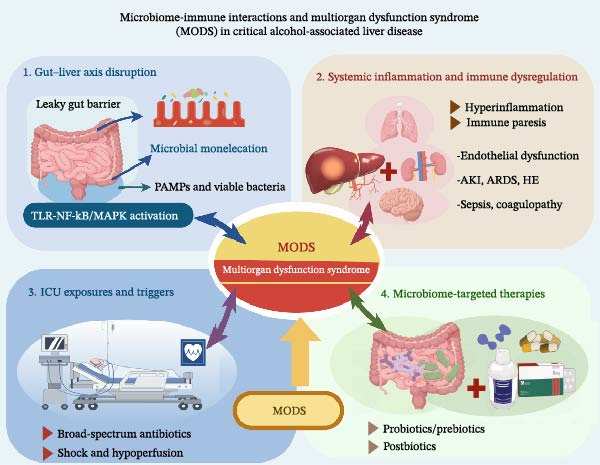
Conceptual framework of the gut–liver axis failure in critical ALD. Critical alcohol‐associated liver disease is depicted as an interconnected pathophysiological network linking gut dysbiosis and barrier disruption, hepatic innate immune activation, intensive care unit‐related secondary hits, and systemic inflammation, culminating in multiorgan dysfunction. These interacting domains shape disease progression, bedside deterioration, and potential therapeutic entry points.

Existing reviews have primarily focused on chronic ALD or selected mechanistic aspects of the gut–liver axis but have less often integrated the ICU‐specific context in which gut barrier failure, systemic inflammation, organ crosstalk, and treatment‐related exposures dynamically interact. In this review, critical ALD refers to ALD presenting with severe AH, acute decompensation, or alcohol‐associated ACLF, requiring ICU‐level monitoring or organ support. Therefore, we aimed to synthesize the current evidence from a critical care perspective, with emphasis on three themes: (i) the evolving role of gut–liver axis failure across the clinical spectrum of severe ALD, (ii) ICU‐related exposures as secondary insults that may exacerbate dysbiosis, barrier disruption, and inflammatory propagation, and (iii) the therapeutic promise and safety limitations of microbiome‐targeted interventions in critically ill patients (Figure 1).

Literature search strategy: This narrative review searched PubMed, Web of Science, and Embase (mainly the past 5 years) using keywords related to ALD, the gut–liver axis, and microbiome therapy, prioritizing human studies (randomized controlled trials [RCTs], cohorts, meta‐analyses, and systematic reviews) and including animal/mechanistic studies only when human evidence was insufficient. Reference lists and relevant reviews were also screened.

## 2. Clinical Spectrum and Temporal Dimensions of Severe ALD in the ICU

### 2.1. Critical ALD Phenotypes and ICU Presentations

The disease spectrum of ALD spans a continuum from steatosis to cirrhosis and hepatocellular carcinoma [1]. However, in critical care, the most relevant phenotypes are those associated with acute deterioration and extrahepatic organ dysfunction, including severe AH, decompensated alcoholic cirrhosis, and alcohol‐associated ACLF [5]. These phenotypes frequently overlap rather than occurring in isolation. For example, severe AH can coexist with cirrhosis [6], acute decompensation may present as systemic inflammation even in the absence of infection [7], and ACLF can be triggered by acute stressors such as infection or bleeding in patients with alcohol‐associated chronic liver disease [8].

Common reasons for ICU admission in patients with critical ALD include sepsis or suspected infection, hemodynamic instability and shock, acute gastrointestinal bleeding, acute kidney injury (AKI), hepatic encephalopathy (HE), and respiratory failure [9, 10]. These presentations often coexist and may evolve rapidly over a short clinical timeframe. Importantly, these ICU manifestations should not be viewed merely as downstream complications of advanced liver disease. Rather, they often represent clinical expressions of gut–liver axis failure, in which dysbiosis, barrier disruption, portal translocation of microbial products, hepatic immune activation, and interorgan crosstalk collectively generate a self‐sustaining inflammatory state that promotes multiple organ dysfunction [11].

### 2.2. Dynamic Evolution of the Gut–Liver Axis and Bedside Decision Points

The pathophysiological role of the gut–liver axis evolves throughout the course of ALD. In the early stages of the disease, alcohol‐induced dysbiosis and mild barrier dysfunction may act primarily as permissive or synergistic factors that contribute to steatosis and low‐grade inflammation. In intermediate stages, including AH and progressive fibrosis, worsening permeability and increased portal delivery of endotoxins and other microbial products amplify hepatic innate immune activation and fibrogenic signaling. In advanced diseases, such as decompensated cirrhosis and alcohol‐associated ACLF, gut barrier injury may progress from increased permeability to functional breakdown, allowing sustained inflammatory translocation that leads to cytokine excess, immune dysregulation, and extrahepatic organ injury [4, 12, 13]. This temporal perspective also helps explain why compensated cirrhosis may remain relatively stable for prolonged periods, whereas acute decompensation can be followed by abrupt inflammatory escalation and organ failure [14, 15].

Risk stratification in critical ALD aims to identify patients at high risk of short‐term mortality and guide time‐sensitive management decisions [16]. In severe AH, liver severity scores, such as the MELD and Lille scores, are often integrated with dynamic response measures to guide decisions regarding the initiation of anti‐inflammatory therapy or evaluation for liver transplantation [17, 18]. In decompensated cirrhosis and alcohol‐associated ACLF, prognostic assessment must also incorporate the multiorgan burden across the circulatory, renal, respiratory, neurological, and coagulation systems [19]. From the perspective of the gut–liver axis, key bedside decision points include infection screening before immunomodulatory therapy, resuscitation strategies aimed at preserving gut perfusion, timing and route of early enteral nutrition, and rational antibiotic stewardship [20, 21]. Thus, clinical assessment is not merely a prognostic tool but also provides actionable nodes for future interventions targeting the gut–liver axis.

## 3. Gut Dysbiosis and Barrier Disruption: An Interacting Pathological Unit

In ALD, gut dysbiosis and barrier disruption are not isolated events but mutually reinforcing processes that convert local intestinal disturbances into systemic inflammation and multiple organ dysfunction [22].

### 3.1. Multilayered Gut Barrier: Coordinated Defense

The intestinal barrier consists of four interdependent components: the epithelial mechanical barrier, mucus and antimicrobial chemical barrier, mucosal immune barrier, and microbial barrier formed by commensal communities [23, 24]. Dysfunction in any one layer may destabilize the others, leading to a feed‐forward pattern of permeability, impaired colonization resistance, and exaggerated inflammatory exposure [25].

### 3.2. Ecological and Functional Changes of Dysbiosis in ALD

Chronic alcohol consumption reshapes the gut microbiota, leading to reduced microbial diversity, depletion of short‐chain fatty acid‐producing commensals, expansion of oral‐origin taxa, and overgrowth of opportunistic pathogens [26, 27]. These ecological shifts compromise short‐chain fatty acid production, bile acid metabolism, and the tryptophan–AhR–IL‑22 protective axis, thereby weakening barrier integrity and promoting endotoxin translocation [28].

### 3.3. Mechanisms of Alcohol‐Induced Gut Barrier Disruption

Alcohol disrupts the intestinal barrier via multiple convergent mechanisms. Ethanol and its metabolite acetaldehyde compromise epithelial tight junction architecture via PP2A‑dependent dephosphorylation and activation of the MLCK pathway [29, 30]. Intestinal CYP2E1‑mediated oxidative stress damages mitochondria and destabilizes junctional proteins [31]. Alcohol reduces the expression of protective antimicrobial peptides, such as Reg3β and Reg3γ, thereby weakening the luminal chemical defense [32]. Disturbed bile acid signaling and impaired FXR‐related pathways compromise barrier maintenance [33]. In addition, alcohol‐associated dysmotility and malnutrition diminish mucosal repair capacity and may predispose critically ill patients to persistent permeability defects [34].

### 3.4. From Permeability to Translocation: Clinical Relevance in Severe ALD

The clinical importance of barrier disruption lies in the sustained translocation of lipopolysaccharides, bacterial DNA, peptidoglycan, and other microbial products into the portal circulation [35]. In ALD, endotoxemia‐related markers, such as lipopolysaccharide‐binding proteins, are elevated compared to those in healthy controls [36]. Translocated microbial signals may contribute not only to hepatic inflammation but also to coagulation activation, endothelial dysfunction, and increased susceptibility to bacterial or fungal infections [37, 38]. In critical ALD, this process is especially relevant because inflammatory translocation may persist despite the temporary stabilization of overt liver injury, thereby sustaining systemic immune dysfunction and interorgan crosstalk [39].

## 4. Hepatic Innate Immune Sensing and Signal Integration: From Multireceptor Networks to Inflammatory Amplification

Once microbial and host‐derived danger signals enter portal circulation, the liver functions as the principal site of signal decoding, amplification, and redistribution [40]. In critical ALD, this hepatic sensing system is exposed to an excessive inflammatory load generated by alcohol‐related barrier failure, impaired clearance capacity, and superimposed stressors.

### 4.1. Portal System: The Gut–Liver Information Pipeline

Portal circulation delivers gut‐derived microbial and metabolic signals directly to the liver [41]. Under physiological conditions, low‐level microbial products are cleared and tolerated [42]. In severe ALD, increased permeability raises the hepatic inflammatory load, whereas impaired liver function reduces the clearance capacity. This mismatch exposes Kupffer cells, sinusoidal endothelial cells, stellate cells, recruited monocytes, and hepatocytes to excessive danger signals, shifting the liver from immune tolerance to inflammatory amplification [43, 44].

### 4.2. Hepatic Innate Immune Sensors: Cellular Players and Receptor Systems

Hepatic innate sensing in ALD is mediated by a coordinated multicellular network involving Kupffer cells, recruited monocytes/macrophages, hepatic stellate cells, liver sinusoidal endothelial cells, and hepatocytes [45]. These cell populations do not act in isolation but instead form an integrated sensing‐and‐amplification system that detects danger signals, produces inflammatory mediators, promotes leukocyte recruitment, and reshapes the local immune tone [46, 47].

At the level of signal integration, the liver relies on a multireceptor network that recognizes both gut‐derived pathogen‐associated molecular patterns (PAMPs) and host‐derived damage‐associated molecular patterns (DAMPs). Toll‐like receptors (e.g., TLR4 sensing LPS and TLR2 sensing peptidoglycan) [48, 49], the NLRP3 inflammasome (activated by reactive oxygen species and extracellular ATP) [50], and nucleic acid sensors such as cGAS–STING and ZBP1 detect distinct but overlapping danger signals [51, 52]. Together, this multireceptor network drives the pathological progression of ALD through synergistic signaling and cascade amplification.

### 4.3. Signal Pathway Synergy, Crosstalk, and Positive Feedback Amplification

A defining feature of hepatic immune activation in ALD is the convergence of distinct sensing pathways on shared downstream inflammatory programs. Alcohol‐related endotoxemia, mitochondrial damage, oxidative stress, and sterile injury signals interact through shared downstream transcriptional and inflammatory programs, generating self‐reinforcing inflammatory circuits [53, 54]. For example, HMGB1/TLR4 signaling may promote NLRP3 and IL‐1β activation, whereas damaged hepatocytes release additional danger signals that further stimulate immune and endothelial cells through TLR4‐ and RAGE‐related pathways [55, 56]. This feed‐forward inflammatory architecture drives the progression from localized hepatic injury to systemic inflammatory dissemination.

## 5. From Liver to Systemic Compartments: Mixed Immune Phenotypes and Multiple Organ Dysfunction (MODS)

### 5.1. Coexistence of Hyperinflammation and Immune Paralysis

Critical ALD is best understood as a mixed immune phenotype rather than a purely hyperinflammatory disorder. Once local containment is exceeded, cytokines, chemokines, extracellular vesicles, and other danger signals spill into the systemic circulation, promoting endothelial activation, oxidative stress, and distant organ injury [57, 58]. Patients may exhibit fever, leukocyte abnormalities, vasoplegia, elevated lactate levels, and capillary leakage, reflecting systemic inflammatory response syndrome [59, 60]. Simultaneously, host defense is compromised through impaired phagocytosis, altered antigen presentation, dysregulated complement and neutrophil responses, and adaptive immune exhaustion [61, 62]. This coexistence of inflammatory activation and functional immune paralysis creates an unstable equilibrium in which pathogen control is suboptimal, yet inflammatory cascades remain self‐sustaining, increasing susceptibility to secondary infections and rapid progression to ACLF and MODS [63]. ALD‐related immune dysfunction is further exacerbated by hepatic insufficiency, altered bile acid signaling, metabolic stress, and malnutrition [64].

### 5.2. Organ–Organ Crosstalk Axes in Severe ALD

**Figure 2:**
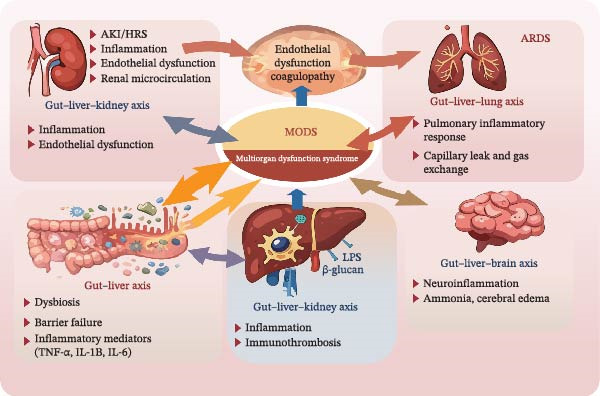
Organ crosstalk network in critical ALD. Gut‐derived inflammatory mediators and microbial products (center) propagate through the gut–liver axis to distant organs, including the kidneys, lungs, and brain. Bidirectional interactions (e.g., lung–gut axis) and systemic inflammation‐coagulation pathways further amplify end‐organ injury. This networked architecture explains why single‐organ support often fails to halt MODS progression.

Systemic inflammation in severe ALD propagates through interconnected gut–liver–kidney, gut–liver–lung, gut–liver–brain, and inflammation–coagulation axes, thereby creating a networked rather than a linear pattern of organ failure. For instance, AKI can precipitate fluid overload and worsen pulmonary edema [65], whereas HE impairs consciousness and elevates the risk of aspiration pneumonia [66]. The major axes are listed in Table 1 (Figure 2).

**Table 1:** Major organ‐crosstalk axes in critical ALD.

Functional axis	Core mechanism	Major clinical manifestations	References
Gut–liver–kidney axis	Endotoxemia and inflammatory mediators promote renal vasoconstriction, tubular injury, and microcirculatory dysfunction in the kidneys.	Acute kidney injury (AKI); hepatorenal syndrome (HRS)	[67, 68]
Gut–liver–lung axis	Systemic inflammation and endothelial injury increase alveolar‐capillary permeability, and mechanical ventilation may further disturb gut perfusion and microbiota.	Pneumonia; acute respiratory distress syndrome (ARDS); refractory hypoxemia	[69, 70]
Gut–liver–brain axis	Gut‐derived ammonia and systemic inflammation (e.g., IL‐1β) disrupt the blood‐brain barrier, activate microglia, and drive neuroinflammation and cerebral edema.	Hepatic encephalopathy (HE); cerebral edema	[71, 72]
Inflammation–coagulation axis	Systemic inflammation triggers endothelial activation and immunothrombosis, whereas impaired hepatic synthesis causes coagulation factor deficiencies.	The coexistence of hypercoagulability with a hemorrhagic tendency; disseminated intravascular coagulation (DIC)	[73, 74]

### 5.3. Why MODS Can Progress Independently of Liver Injury

MODS in critical ALD may progress even after partial control of the initial hepatic insult because systemic inflammatory circuits can become self‐sustaining [75, 76]. Circulating mediators persistently activate endothelial and immune cells [77], while injured distant organs release DAMPs that further amplify sterile inflammation, independent of ongoing hepatic involvement [78]. This vicious cycle, compounded by immune paralysis and metabolic exhaustion, explains why abrupt deterioration, culture‐negative inflammatory syndromes, and high mortality from superimposed infections are common in critically ill patients with ALD [79, 80].

## 6. ICU Exposure as a Second Hit to the Gut–Liver Axis

In critically ill patients with ALD, ICU care is not biologically neutral. Life‐saving interventions may also act as secondary hits to the gut–liver axis, reshaping the microbial ecology, impairing barrier integrity, reducing gut perfusion, and sustaining inflammatory translocation. These effects are particularly relevant for broad‐spectrum antibiotics, acid suppression, parenteral nutrition, vasopressor‐dependent shock, sedatives or opioids, and mechanical ventilation [81] (Table 2).

**Table 2:** ICU‐related secondary hits to the gut–liver axis and their clinical implications in critical ALD.

ICU exposure factor	Primary impact on gut–liver axis	Clinical implication	Reference
Broad‐spectrum antibiotics	Loss of diversity, commensal depletion, resistant pathogen overgrowth, reduced SCFAs	De‐escalation therapy; limit duration; avoid routine prophylactic antifungals	[82, 83]
Acid suppression (PPI)	Loss of gastric acid barrier, oralization of gut microbiota, increased translocation risk	Strict indications and clear withdrawal criteria	[84, 85]
Parenteral nutrition	Disuse atrophy of intestinal mucosa. Reduced secretion of antimicrobial peptides	Prioritize enteral nutrition, fiber supplementation, and postpyloric feeding	[86, 87]
Shock/vasopressors	Intestinal ischemia‐reperfusion injury. Epithelial apoptosis/shedding, tight junction disruption, and acutely increased permeability	Rapid restoration of perfusion; avoid excessive vasoconstriction	[88, 89]
Opioids/sedatives	Gut dysmotility, bacterial overgrowth, impaired mucosal repair	Multimodal analgesia; early prokinetic intervention	[90, 91]
Mechanical ventilation	Intestinal wall congestion and edema, gut dysbiosis, elevated intra‐abdominal pressure reducing visceral perfusion	Protective lung ventilation; reduction of intra‐abdominal pressure	[92, 93]

Even with optimized ICU management, dysbiosis and barrier injury may persist in critically ill patients, allowing continued endotoxin translocation and inflammatory propagation [94]. This provides a rationale for considering microbiome‐targeted interventions, which will be discussed in the next section, with an emphasis on safety boundaries and clinical applicability.

## 7. Microbiome‐Targeted Therapy: Key Evidence, Safety Margins, and Precision Application Strategies

Because microbial translocation and barrier failure contribute to hepatic and systemic inflammation, microbiome‐targeted therapies are biologically attractive in patients with severe ALD [95]. As summarized in Table 3, probiotics, prebiotics, synbiotics, postbiotics, and fecal microbiota transplantation have each demonstrated benefits in selected contexts—ranging from improvements in liver enzymes and microbial composition to enhanced short‐term survival in severe AH. However, clinical translation remains limited by heterogeneous trial designs, small sample sizes, variable products and dosing, limited ICU‐specific evidence, and a lack of standardized mechanistic endpoints [110, 111].

**Table 3:** Key clinical evidence and safety considerations for microbiome‐targeted therapies in alcohol‐associated liver disease.

Microbiota‐targeted therapy	Core mechanism	Main clinical evidence and findings	Major safety concerns
Probiotics	Restore gut barrier, suppress inflammation, modulate flora and lipid metabolism	Meta‐analyses (2024–2025, 5–12 RCTs): Probiotics significantly improved liver function (ALT, AST, and GGT levels) and modulated the gut microbiota in ALD; however, one analysis noted no significant improvement in inflammatory markers [96–98]. RCT (2025, *n* = 42 ALD): 30‐day multistrain probiotic reduced ALT, AST, and GGT levels and remodeled microbial/metabolic profiles [95].	Risk of bacteremia/fungemia (increased intestinal permeability, central venous catheters, and immunodeficiency) and strain heterogeneity [99].
Prebiotics	Selectively stimulate beneficial bacteria, increase SCFAs, restore gut homeostasis	RCT (2022, *n* = 50 alcohol use disorder): Inulin during abstinence did not improve liver function and was associated with elevations in AST, ALT, and IL‐18 [100]. Animal models: pectin‐derived prebiotics prevent alcohol‐induced liver injury in murine models [101].	Use with caution in ileus/severe diarrhea; excessive intake may cause bloating [102].
Synbiotics	Synergistic interactions between probiotics and prebiotics.	In a small study (*n* = 10 alcoholic cirrhosis + 10 NASH), 2‐month synbiotic intervention improved ALT and GGT; however, the benefits disappeared after a 1‐month washout [103].	Interindividual variability; dependency on component ratio/dosage; long‐term safety unknown [104].
Postbiotics	Direct effects of microbial metabolites or inactivated components	Phase 2 RCT (2025, *n* = 56, advanced ALD): 12‐week postbiotic ReFerm did not meet the primary endpoint (liver fibrosis improvement). However, it was safe and showed secondary benefits, such as reduced liver injury markers and increased fecal SCFA [105].	Theoretically safer than live biotherapeutics; optimal components/dosing need to be defined [106].
Fecal microbiota transplantation (FMT)	Infusion of healthy donor microbiota to reconstruct gut ecosystem	RCT (2023, *n* = 120 severe ALD): FMT improved 90‐day survival (75% vs. 56.6% for prednisolone) and reduced infection‐related mortality (from 19.3% to 3.6%) [107]. Meta‐analysis (2025, 8 studies, *n* = 444 sAH): FMT significantly improved 28‐day and 90‐day survival, with no serious adverse events reported [108].	Pathogen transmission, exacerbation of inflammation, bacteremia, and stringent donor screening are required [109].

### 7.1. Safety Margins: When Microbiome Therapy Becomes High‐Risk in the ICU

**Figure 3:**
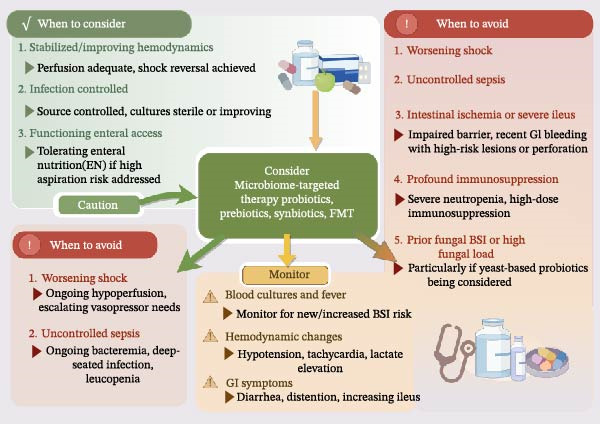
Safety‐oriented decision framework for microbiome‐targeted therapies in ICU patients with critical ALD. Before initiating probiotics, postbiotics, or fecal microbiota transplantation, patients should be evaluated for major high‐risk features, including severe intestinal barrier disruption, invasive vascular devices, profound immune dysfunction, uncontrolled infection, and a high fungal burden. Microbiome‐directed therapy should be considered only in selected stabilized patients and requires close monitoring for fever, bloodstream infection, and hemodynamic deterioration after treatment initiation.

Available studies suggest the potential benefits of microbiome‐targeted approaches in ALD; however, direct extrapolation to critically ill patients carries a substantial risk [112]. In critical ALD, severe barrier disruption, central venous catheters or other invasive devices, uncontrolled infection, profound immune dysfunction, and high fungal burden may increase the risk of bacteremia, fungemia, pathogen transmission, and inflammatory deterioration [113–115]. A pragmatic ICU approach is to consider microbiome‐targeted therapy only in selected stabilized patients with improving perfusion, controlled infection, and functioning enteral access [116]. These interventions should be avoided or deferred in patients with ongoing shock, uncontrolled sepsis, intestinal ischemia, severe ileus, active or recent gastrointestinal bleeding, or unexplained hemodynamic instability [117]. After initiation, clinicians should monitor for new fever, bloodstream infections, worsening shock, and temporal associations with specific products, strains, or dosing regimens [118] (Figure 3).

### 7.2. Toward Precision Application: Matching Therapy to Phenotype and Endpoints

Given this heterogeneity, the field will likely progress through phenotype‐informed strategies rather than through empirical supplementation. For example, barrier‐dominant phenotypes with high permeability or endotoxemia may be more suitable for postbiotics, prebiotics, or gut‐protective nutrients [119]. Infection‐prone phenotypes with recurrent sepsis may require strategies aimed at restoring colonization resistance but only after rigorous safety screening [120]. Encephalopathy‐dominant phenotypes may benefit from integrating microbiome modulation with standard HE therapy and motility management [121]. Future trials should therefore combine mechanistic biomarkers, ICU exposure data, safety endpoints, and clinically meaningful outcomes.

### 7.3. Controversy: Alcohol‐Associated Liver Inflammation Is Not Strictly Dependent on Gut Translocation

Although gut‐derived microbial translocation is a major amplifier of inflammation in severe ALD, alcohol‐associated liver injury is not exclusively dependent on the gut–liver axis. Alcohol and its metabolites can directly injure hepatocytes through oxidative stress, mitochondrial dysfunction, endoplasmic reticulum stress, and nonclassical cell death pathways. Injured hepatocytes can also initiate sterile inflammatory loops through the release of DAMPs, such as HMGB1 [122–124]. Concurrently, alcohol‐driven oxidative stress modifies hepatic epigenetic regulation independently of translocation, including epigenetic dysregulation [125]. Furthermore, in vitro and in vivo studies have confirmed direct Kupffer cell activation, with alcohol significantly inducing TNF‐α and IL‐6 expression in these macrophages, further exacerbating hepatocellular injury and inflammation [126]. Therefore, ALD pathogenesis arises from a combination of direct alcohol hepatotoxicity and intrahepatic innate immune activation. These gut‐independent mechanisms are intertwined with the indirect amplifying effects of the gut–liver axis.

## 8. Conclusion and Future Perspectives

Severe ALD should not be understood merely as advanced hepatic injury but as a systemic inflammatory disorder centered on gut–liver axis failure. Alcohol‐induced dysbiosis and intestinal barrier disruption enable sustained translocation of microbial and host‐derived danger signals to the liver, where a multireceptor innate immune network amplifies the inflammatory output and promotes the progression from local liver injury to systemic immune dysregulation and multiple dysfunctions. In the ICU, this trajectory may be further intensified by secondary hits, such as antibiotics, acid suppression, parenteral nutrition, shock, and mechanical ventilation. Microbiome‐targeted therapies offer a promising upstream strategy; however, their use in critically ill patients must remain phenotype‐informed and safety‐conscious. Future advances will depend on integrating mechanistic biomarkers, multiomics profiling, and artificial intelligence‐based risk models to define inflammatory phenotypes, therapeutic windows, and individualized intervention pathways in patients with critical ALD.

Future progress is likely to depend on the integration of inflammatory biology with precision risk stratification. Emerging biomarkers, proteomic signatures, multiomics approaches, and AI‐based prediction models may help identify dominant inflammatory phenotypes, forecast short‐term outcomes, and guide the selection of microbiome‐targeted or other mechanism‐based interventions. Several recent examples illustrate this potential in the literature. A plasma urobilinogen level >0.07 mg/mL accurately predicts steroid nonresponse and early mortality [127]. Complement protein signatures independently forecast 90‑day mortality risk [128]. Large‑scale proteomic profiling enables efficient distinction of MetALD subtypes using only 10 plasma proteins (AUC 0.93) [129]. The AI model ALCHAIN, developed from global multicenter cohorts, predicts 90‑day mortality in AH more accurately than conventional scores (AUC 0.799) [130]. These tools should not be viewed as alternatives to the gut–liver axis framework but as strategies for identifying when, in whom, and under what safety conditions gut‐targeted interventions are most likely to succeed.

## Funding

This study was supported by the National Natural Science Foundation of China (Grants 82460373 and 82560382), the Zunyi Science and Technology Bureau, China (Grants HZ (2025) 312, HZ (2023) 366 and HZ (2025) 172), the Guizhou Administration of Traditional Chinese Medicine (Grant QZYY‐2024‐137), the Guizhou Provincial Health Commission (Grant gzwkj2024‐310), the Kweichow Moutai Hospital Research Project (Grant MTyk2024‐55), and the Science and Technology Development Fund of the Affiliated Traditional Chinese Medicine Hospital of Zunyi Medical and Pharmaceutical College (Key Project) (Grant HZ202411).

## Conflicts of Interest

The authors declare no conflicts of interest.

## Data Availability

Data sharing is not applicable to this article, as no datasets were generated or analyzed during the current study.
